# Periscope of Dermatology

**Published:** 1895-08-24

**Authors:** 


### Periscope of Dermatology.

**Lupus.**—Hutchinson[1] describes a case of lupus on the chest which originated in a congenital mole or nævus. He considers that lupus does not arise by inoculation of the tubercle bacillus, but that the parasite is present in the tissues at birth, and any weak spot favours its development; the structure of a nævus may be more vulnerable than healthy skin.

Abraham[2] describes a woman, aged 50, with a patch of lupus vulgaris on the face. At first it had resembled syphilis, but had not yielded to antisyphilitic treatment. It was now improving under thyroid feeding.

Hill[3] describes a case of lupus of the soft palate and nose, which he was treating with local applications of lactic acid, and internally with grey powder.

Cæsar[4] refers to two cases of lupus treated by excision, in neither of which had there been any recurrence. He strongly recommended this treatment.

Cahill[5] records a case of tubercular lymphangitis, which commenced from the irritation of a corn or wart at the back of the heel. The foot and ankle were swollen, and there was an irregular chain of firm nodular masses of a deep red colour, which was most marked on the heel and instep, but extended irregularly up the back of the leg. No tubercle bacilli were found, but the disease was considered to be lupus. Pringle[6] also quotes similar cases.

Purdon[7] reports the case of a woman, aged 24, who suffered from tuberculous lymphangitis. On the dorsum of the toes and foot there were several firm nodular masses which were quite painless; on either side of the ankle there were rough warty patches resembling lupus verrucosus, and on the dorsum of the second toe there was a similar patch, which was ulcerated; on the face there was a typical eruption of lupus vulgaris.

Thibaudet[8] publishes a case of lupus verrucosus whose origin was distinctly due to local infection. A woman, aged 40, had slightly wounded the back of her hand while making the bed for a phthisical patient. The wound did not heal, and eighteen months later a patch of lupus verrucosus appeared in the scar, and reached the size of a two-franc piece. The author notes that the warty form of lupus is that usually observed after professional inoculation.

Stowers[9] describes a case of lupus erythematosus occurring in a girl, aged 18 years, in which a perfectly smooth scar resulted after linear scarification.

Audry[10] records a case of lupus erythematosus in which the patient died from pulmonary disease without the presence of tubercle bacilli in the sputum. Microscopical examination of the skin lesions showed tubercular infiltration with giant cells, but no tubercle bacilli.

Brooke,[11] although denying that all cases of lupus erythematosus owe their origin to tubercle, considers that in some, at least, there is evidence of the co-operation, at any rate, of the tubercle bacilli in their genesis. He quotes a case in which lupus erythematosus was associated with abdominal tuberculosis and in which the former disease seemed to be distinctly influenced, if not directly conditioned, by the latter. In this case the lupus extended rapidly, while the abdominal trouble was advancing. After death no tubercle bacilli were found in the skin.

**Eczema.**—Lockwood[12] records a case of eczema capitis which had existed four years in spite of various treatment, but which was quickly cured after the internal administration of salicylate of soda. Hutchinson[13] states that eczema and psoriasis are liable to hereditary transmission, but that one disease in the parent may appear as the other in the child. He instances the case of a mother and daughter who had eruptions in the same situations, the mother having scaly eczema, the daughter having psoriasis.

Unna[14] considers that the eczema on the hands and fingers of the poor is allied to seborrhoic eczema, and that there is usually some pityriasis capitis present at the same time which ought to be treated. Locally he rubs the hand with green soap and water, and applies a paste over which strips of the thinnest gutta-percha tissue are firmly bound. This is rubbed off when the day's work is done, and a fresh dressing applied at night. The following pastes are recommended: (1) ℞. zinc oxide, 40 parts; cretæ præparata; liq. plumbi subacetat. dil., ol. lini, a.a., 20 parts; m. (2) Oxide of lead, 50 parts; vinegar, 75 parts; linseed oil, 25 parts, m. (3) Equal parts of oxide of lead, zinc sulphate, chalk, linseed oil, and lime water, are shaken together to form an emulsion. In washerwomen resorcin is added, so as to strengthen the epidermis. Schwemmer[15] groups the causes of eczema into (1) local and (2) general; he sub-divides the latter into (*a*) constitutional; (*b*) nerve influences; and (*c*) micro-organisms. Local irritation can only produce true eczema in persons predisposed to the disease. He considers that the nervous system has the greatest influence in the production of the disease, and disputes the influence of micro-organisms. Escheverria[16] describes the histology of epidemic dermatitis. He considers that the most important changes in this disease are in the nuclei of the prickle cells; there is general hypertrophy in the lower, and shrinking in the upper strata. He considers that histologically the disease is totally different from chronic eczema.

Carrucio[17] records a case of eczema impetiginosa in which staphilococcus aureus was cultivated from the unbroken pustules, and also from the urine. He considered that the skin affection was caused by infection from the kidneys.

**Psoriasis.**—Seifert[18] records thirteen cases of psoriasis treated with large doses of iodide of potassium. He recommends this treatment as an adjunct to external remedies, and considers it of value in those cases where the general health is good, and where the urine is free from albumen.

Coffin,[19] in moderate cases of psoraisis, removes the scales with an alkaline bath, and applies the following preparation; Glycerolate of starch, oil of juniper, a.a., ℥3½; green soap, gr. 75; and acid salicylic, gr. 45, m. In obstinate cases the following salve is used: Ichthyol, salicylic, and pyrogallic acids, a.a., gr. 30; vaseline, lard, and lanoline, a.a., ℥1. Arsenic is only given when the disease has ceased to progress.

**Herpes Zoster.**—Douglas[20] records a case of bilateral herpes zoster of the fifth pair of nerves. Patches of vesicles appeared at the points of exit from the bony canals of each branch of these nerves. There was severe neuralgia.

Drinkwater[21] relates a case of herpes, which followed the distribution of the superficial cervical plexus. Great amelioration of the neuralgic pain occurred after the internal administration of camphor.

Wingfield,[22] from examination of the blood of patients suffering from herpes zoster, demonstrated the presence of the plasmodia malaria. He therefore considers that malaria can and does produce herpes.

Hartzell[23] found bodies, resembling protozoa, in the vesicles of herpes. Some had a double contour and contained two nuclei, others resembled large epithelial cells, and a third kind were pear-shaped and contain coccydia.

Kaposi[24] finds the following ointment most soothing during the ulcerating stage of the disease: ℞. boric acid gr. 1, glycerine q.s., vaseline grs. 30, cocaine hydrochlorate, extract of opium a.a. centigr. 30, m. The neuralgia following the eruption is best treated by Fowler's solution.

**Parasitic Diseases.**—In a clinical lecture on the itch, Leloir[25] gives a full account of the life history of the acarus, and states that from an objective point of view scabies is characterised by its polymorphous eruption. He divides the eruptions into two kinds which are distinct from each, pathologically and etiologically; (1) the acaric eruption, properly so-called, produced by the burrowing of the female acarus, and (2) the quasi scabieic dermatoses. The latter are further divisible into (*a*) quasi scabieic dermatoneuroses, (*b*) quasi scabieic dermites, and (*c*) quasi scabieic pyodermites. Group (*b*) is further subdivided into (1) eczematiform dermites, and (2) lichenification of the integument. The polymorphic eruption, situated, in the first instance, on special cutaneous surfaces, and abstaining for a time from invasion of the remainder of the integument, imposes upon the malady a peculiarly distinctive character.

Eddowes[26] reports a case of eczema impetiginodes of parts of the fingers of both hands, which occurred in a student of Asiatic languages. He traced the infection of the fingers to the manuscript at which the patient was working. He refers to cases of eczema of the leg, which were caused by infection from the other leg, by interchanging the stockings.

Leistikow[27] recommends the following treatment for sycosis dependent on staphylococci: In slight cases he applies a sulphurated zinc paste to which a 5 per cent. solution of carbolic acid has been added, and in severe cases he advises cauterisation with a 20 to 50 per cent. solution of resorcin in alcohol. In the most rebellious cases he uses an ointment of pyrogallol or of chrysorobin.

Cantrell[28] recommends that a solution of salol in petrolatum (twenty grains to the ounce) should be applied twice daily in cases of sycosis, and quotes several cures.

Zinsser,[29] having found that the growth of favus fungus was killed by a temperature of 45 C., treated five cases of favus by means of heat, which was applied by passing water, at a temperature of 113 Fahr., through a properly fitted coil on the head, beneath which was a compress saturated with a 1 in 2,000 solution of bichloride of mercury. The heat was applied continuously for twelve hours, and in all but one case a cure resulted within a short time.

Van-Hoorn[30] recommends the following treatment for boils: The skin is first aseptised by washing, and by a 1 in 1,000 solution of mercury bichloride; the area of the boil is then completely covered with a phenol and mercury plaster which is changed daily. If the boil bursts it is cleaned out with a strong antiseptic, and cicatrisation is very rapid.

Brocq,[31] having noted that gouty people are often attacked by successive crops of boils, now treats such cases by the internal administration of colchicum. In one case the boils quickly disappeared under the influence of the colchicum, but reappeared when the drug was stopped. Locally he applies strong tincture of camphor.

**Angio-Neurotic Œdema.**—Smith[32] reports the case of a woman aged twenty-four, undoubtedly of a neurasthenic type, who suffered from periodical swellings of the whole body, which came on before or after each period. They were accompanied by a feeling of heat all over the body and by itching. Mental excitement and worry seemed to increase the swellings.

Evans[33] relates the case of a woman aged thirty years, who, after each confinement, suffered from excessive swelling of the body, face, and limbs, accompanied by extreme collapse and followed by flooding. After her last confinement she also suffered from neuritis of the left facial, musculo spiral, and median nerves. She also had symptoms of Grave's disease. Evans considers this to be a case of angio-neurotic œdema.

**Tumours.**—Jamieson[34] records an interesting case of xanthoma diabeticorum occurring in a man aged fifty-five. The eruption was not found on the elbows, knees, or buttocks, and the colour was unusual, since there was an entire absence of distinct yellow. Its relation to glycosuria was indisputable.

Hutchinson[35] comments on a similar case.

Lassar[36] notes the different prognosis in cases of melanoma according as to whether the growth is carcinomatous or sarcomatous. The former appear to be less likely to recur after free removal than the latter. He also quotes the case of a woman aged thirty, who developed a melanoma beneath the breast in the site of a pigmented mole; this shrunk up under treatment with arsenic.

Zumbusch[37] describes a case of granuloma fungoides occurring in a man aged seventy-three years. The thickening of the skin, together with the exaggeration of the folds, gave his face the appearance of tubercular leprosy. The condition had existed for two years and had been preceeded during four years by attacks of skin eruptions of an eczematous or erythematous type. There were no circumscribed or fungating tumours, but there was round-celled infiltration of the corium and subcutaneous tissue. He considers that the tumours would have developed had the patient lived long enough.

[1] Clin. Journ., Feb. 26, 1895, p. 275. [2] Ditto, April 3, 1895. [3] Ditto, ditto. [4] Lancet, April 6, 1895, p. 869. [5] Brit. Journ. of Derm., Jan., 1895, p. 1. [6] Ditto, ditto, p. 4. [7] Ditto, March, 1895, p. 80. [8] Ditto, Jan., 1895, p. 34. [9] Clin. Journ., Feb. 27, 1895, p [illegible]. [10] Ed. Med. Journ., April, 1895, p 950. [11] Brit. Journ. of Derm., March, 1895, p. 73. [12] Med. Rev. Cord., Feb., 1895, p. 216. [13] Clin. Journ, Feb. 13, 1895, p. 259. [14] Ed. Med. Journ., June, 1895, p. 1,129. [15] Brit Journ. of Derm., Jan., 1895, p. 33. [16] Ditto, ditto, p. 9. [17] Ditto, March, 1895, p. 96. [18] Ditto, April, 1895, p. 130. [19] Ditto, May, 1895. p. 167. [20] Brit. Med. Journ., April 13, 1895, p. 808. [21] Ditto, ditto, p. 809. [22] Med. Rec., Feb. 16, 1895. [23] Int. Med. Mag., Feb., 1895; Brit. Journ. of Derm., March. 1895, p. 99. [24] Med. Week, April 5, 1895. [25] Prov. Med. Journ., March 1, 1895. p. 139. [26] Brit. Med. Journ., March 2, 1895, p. 471. [27] Les Nouv. Remed., March, 1895. [28] Ther. Gaz., Feb. 15, 1895. [29] Am. Med. Surg. Bull., March 1, 1895. p. 363. [30] Ind. Med. Chir. Review, March 30, 1895. [31] New York Med. Journ., March 30, 1895. [32] Am. Med. Surg. Bull., Feb. 1, 1895, p. 178. [33] Lancet, June 1, 1895, p. 1,375. [34] Glas. Med. Journ., May 8, 1895, p. 317. [35] Clin. Journ., May 8, 1895, p. 29. [37] Brit. Med. Journ. Ep., May 4, 1895, No. 338.

